# Supplementary figures and images for: Multi-omics analysis identifies loci associated with pyrethroid resistance across sister species in the Anopheles gambiae species complex
Source: BMC Genomics. 2026 Jul 7;27:594. doi: 10.1186/s12864-026-13109-8 (PMC13339448; doi:10.1186/s12864-026-13109-8)

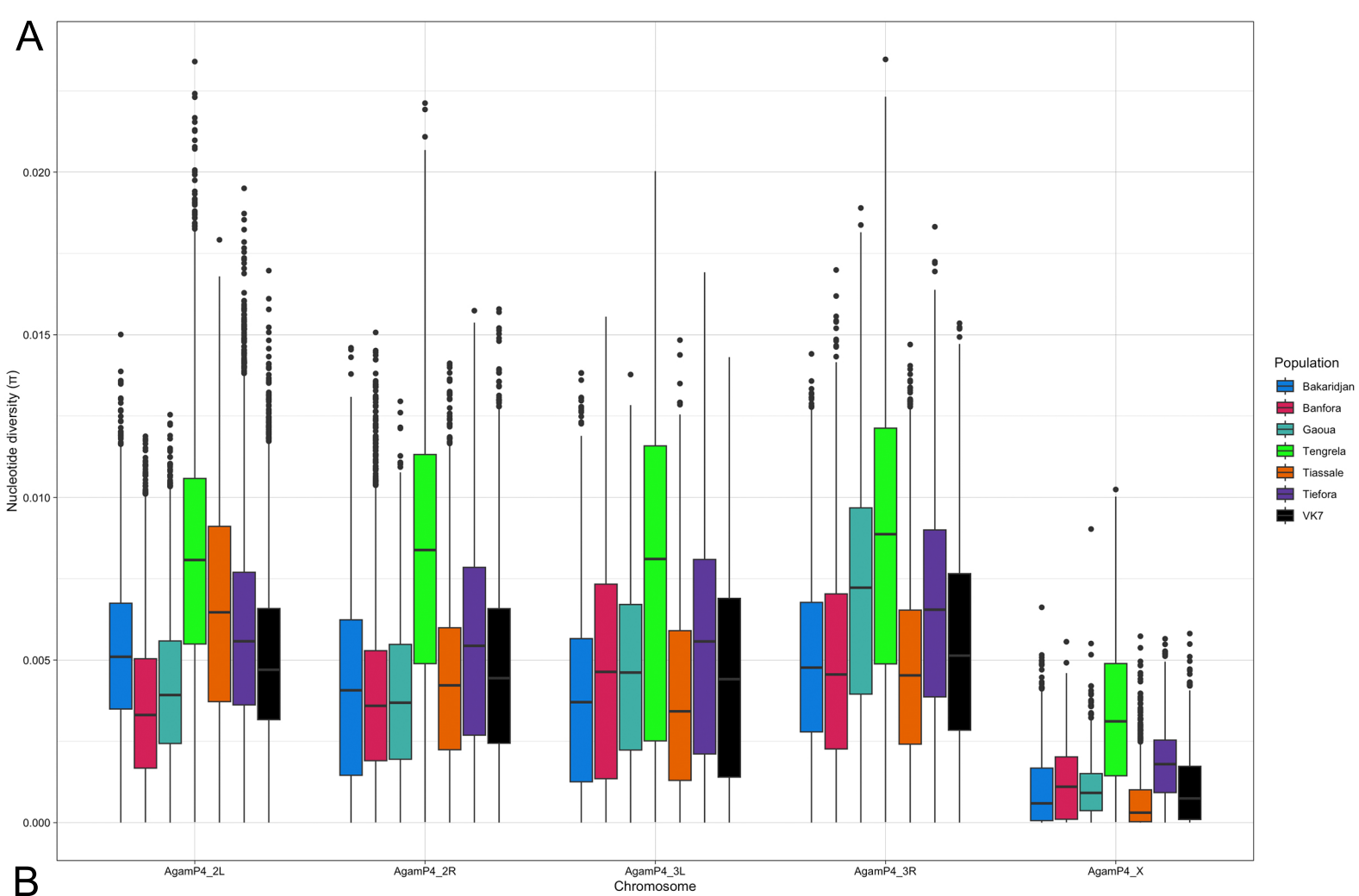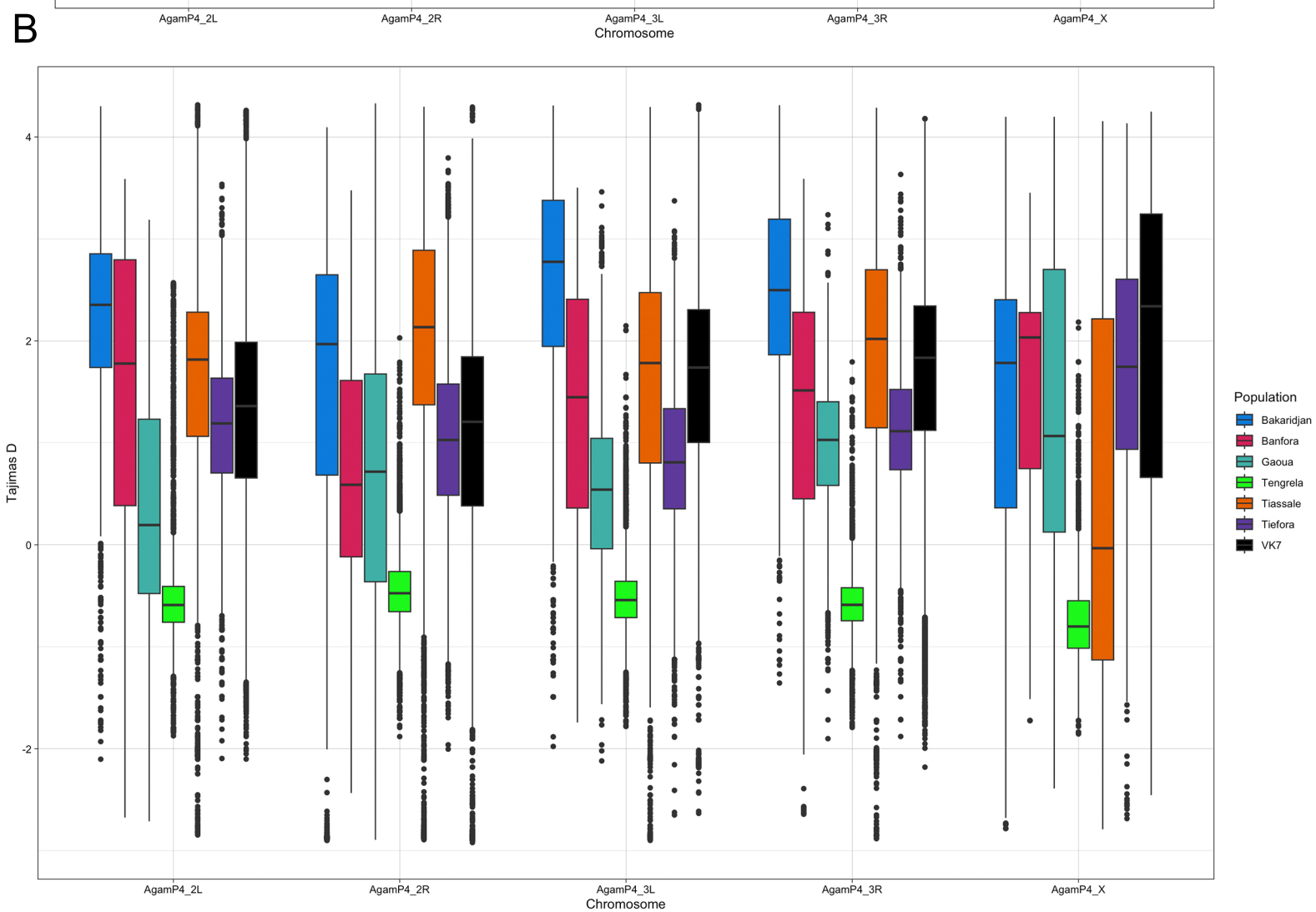

Supplement: Supplementary file 1 — Supplementary Material 1. Supplementary Figure 1: Descriptive statistics for each population. A. Nucleotide diversity and B. Tajimas D statistic for each population sequenced. The respective statistics are shown on the y axis and the x axis shows individual chromosomes. Each box plot represents a separate population, coloured as in the key. [file 12864_2026_13109_MOESM1_ESM.pdf]

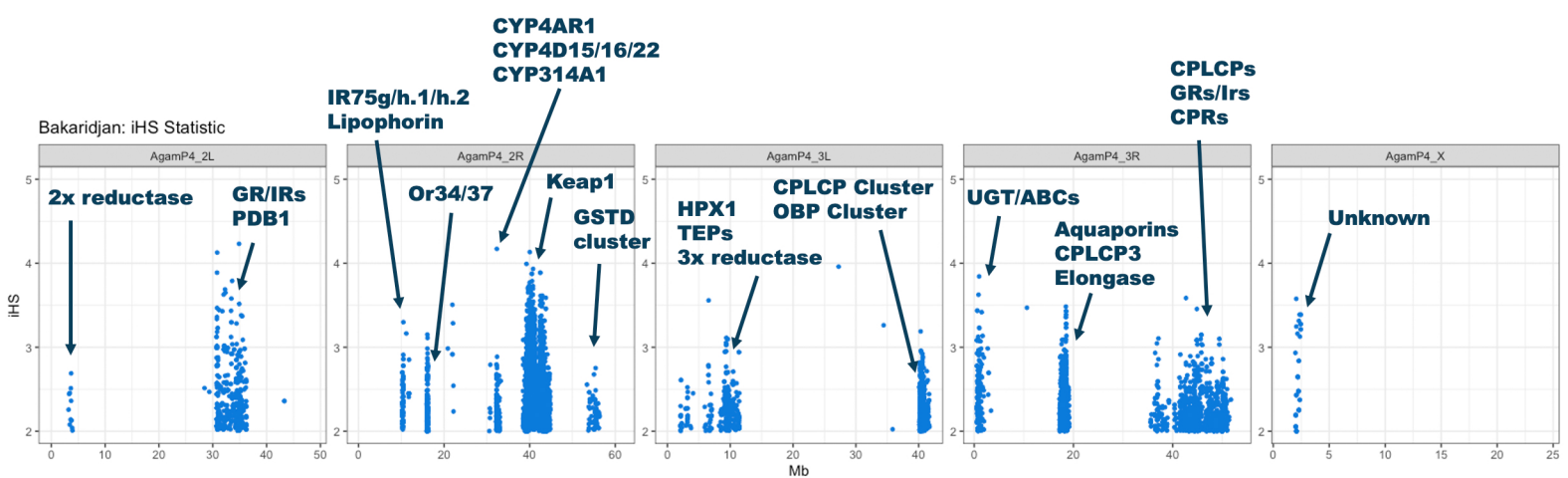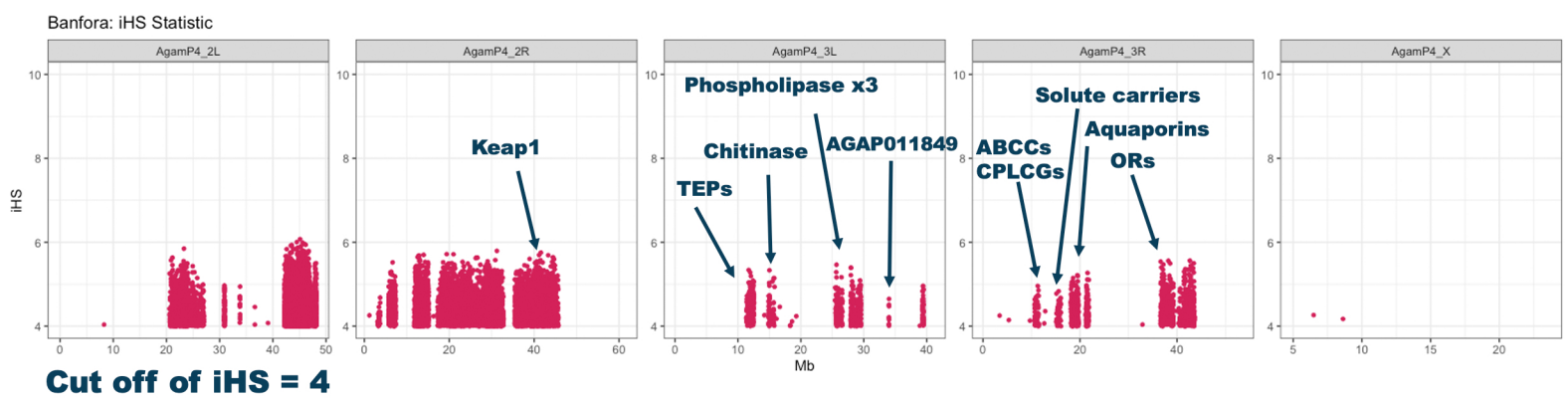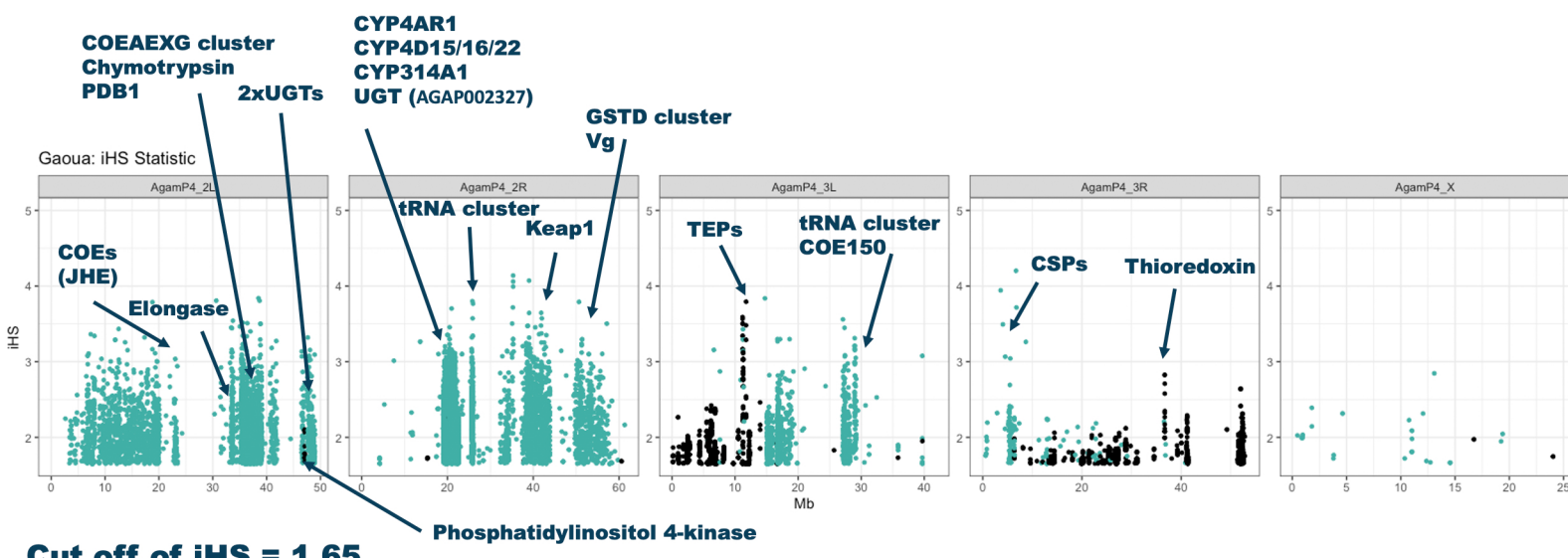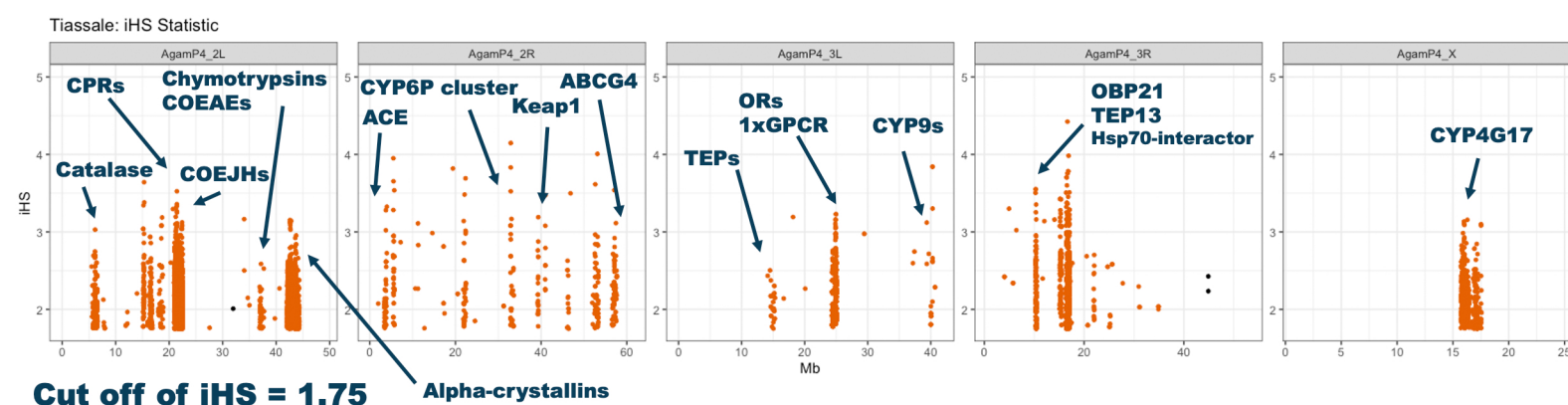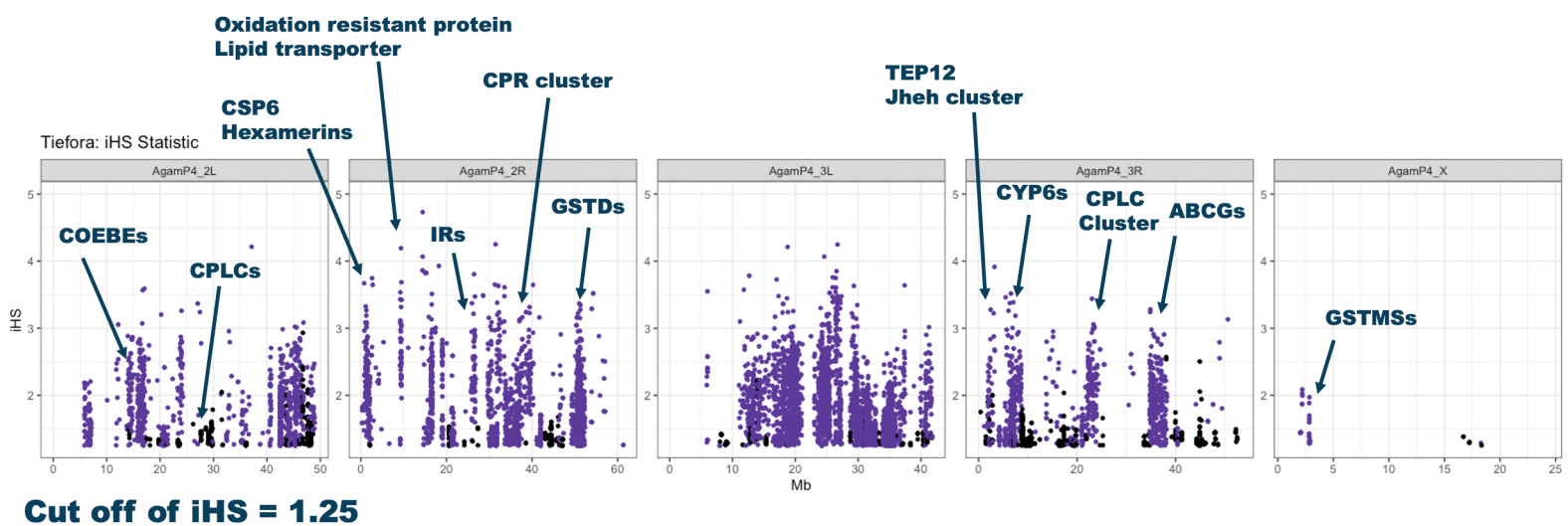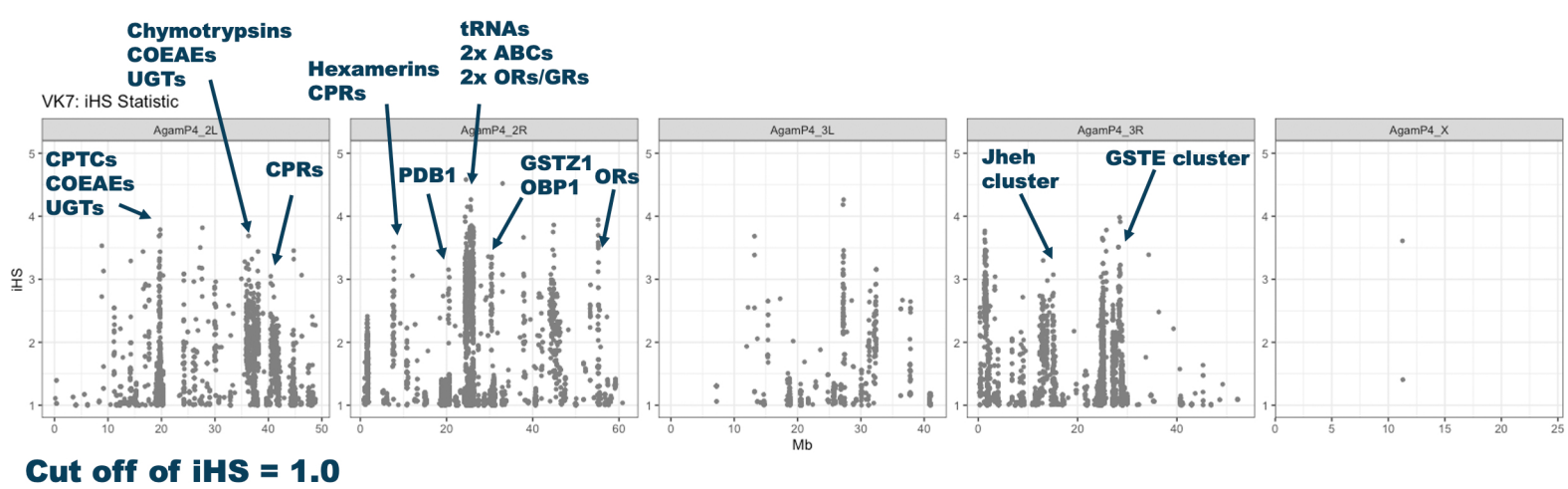

Supplement: Supplementary file 2 — Supplementary Material 2. Supplementary Figure 2: Absolute iHS statistic for the colony populations. iHS statistic (y axis) for the length of each chromosome (x axis) as indicated at the top of each panel. Colonies are coloured as previously, and the title of the graph indicates the colony. Displayed are the top 5 % of the statistic only, cut-off indicated below. Negative iHS are shown in black. Labels correspond to putative insecticide-resistant associated transcripts in regions of elevated iHS. [file 12864_2026_13109_MOESM2_ESM.pdf]

**A**

kdr

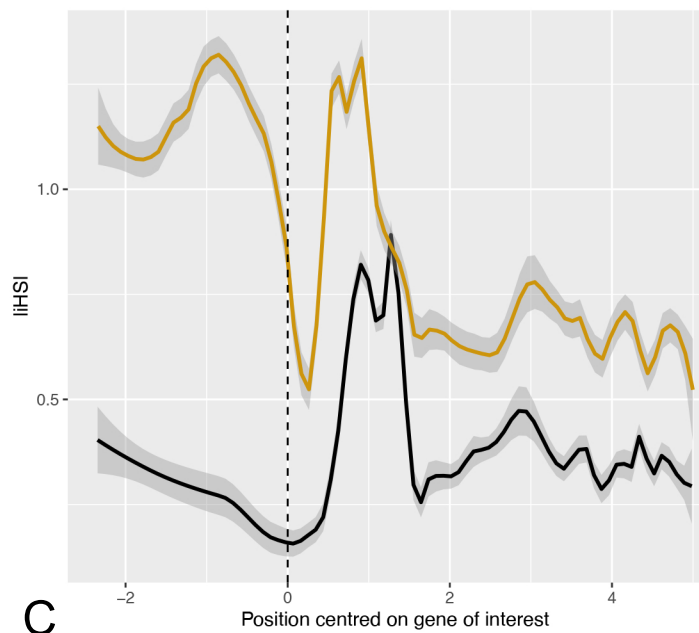**B**

CYP6P3

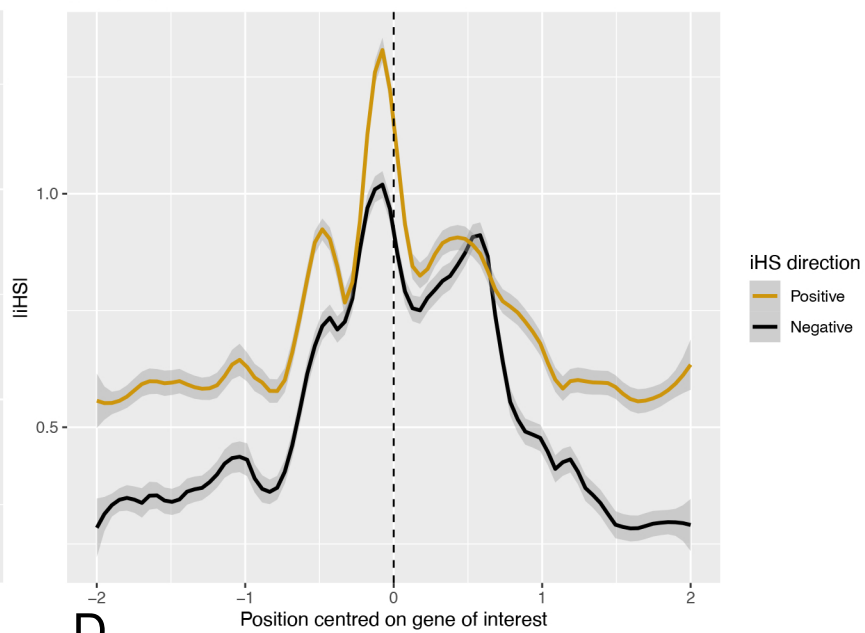**C**

CYP9K1

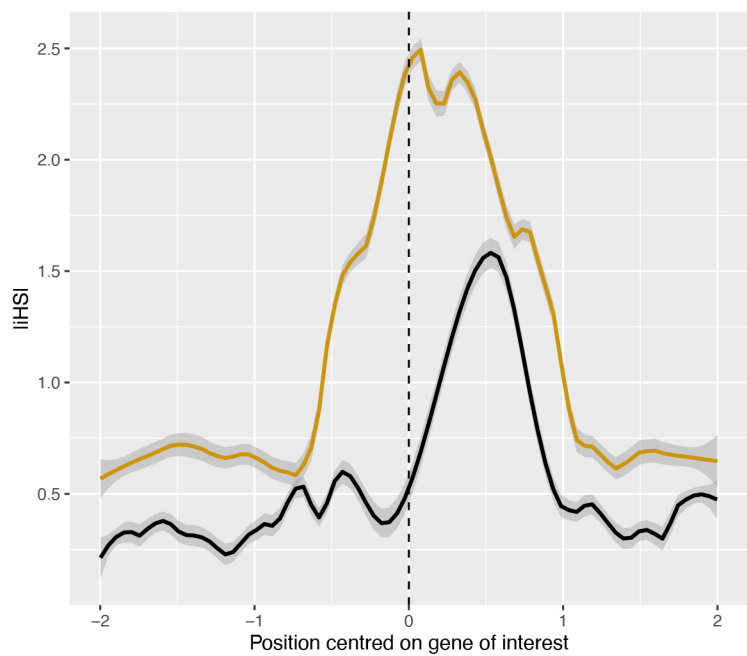**D**

GSTE2

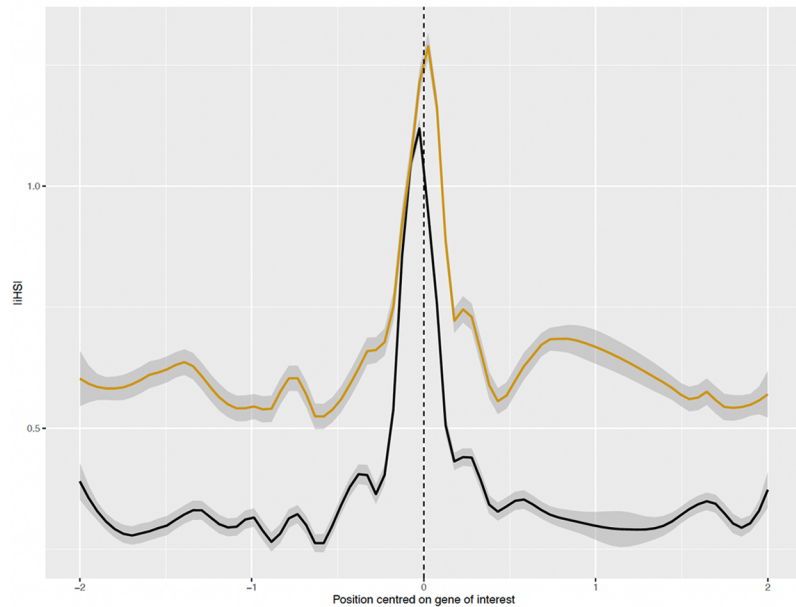

Supplement: Supplementary file 4 — Supplementary Material 4. Supplementary Figure 4: iHS sweeps in the Tengrela (F) population centred on genes of interest. Absolute iHS statistic (y axis) for each resistance-related sweep location in the field-caught Tengrela (F) population. The loci of the genes of interest were taken as the centre point +/- 2Mbp (x axis). The dotted line represents the gene of interest: A. kdr, B. CYP6P3, C. CYP9K1 and D. GSTE2. The yellow line represents positive iHS and black negative. Negative values signify selection on derived alleles while positive values are associated with selection on ancestral alleles. [file 12864_2026_13109_MOESM4_ESM.pdf]

GSTE2

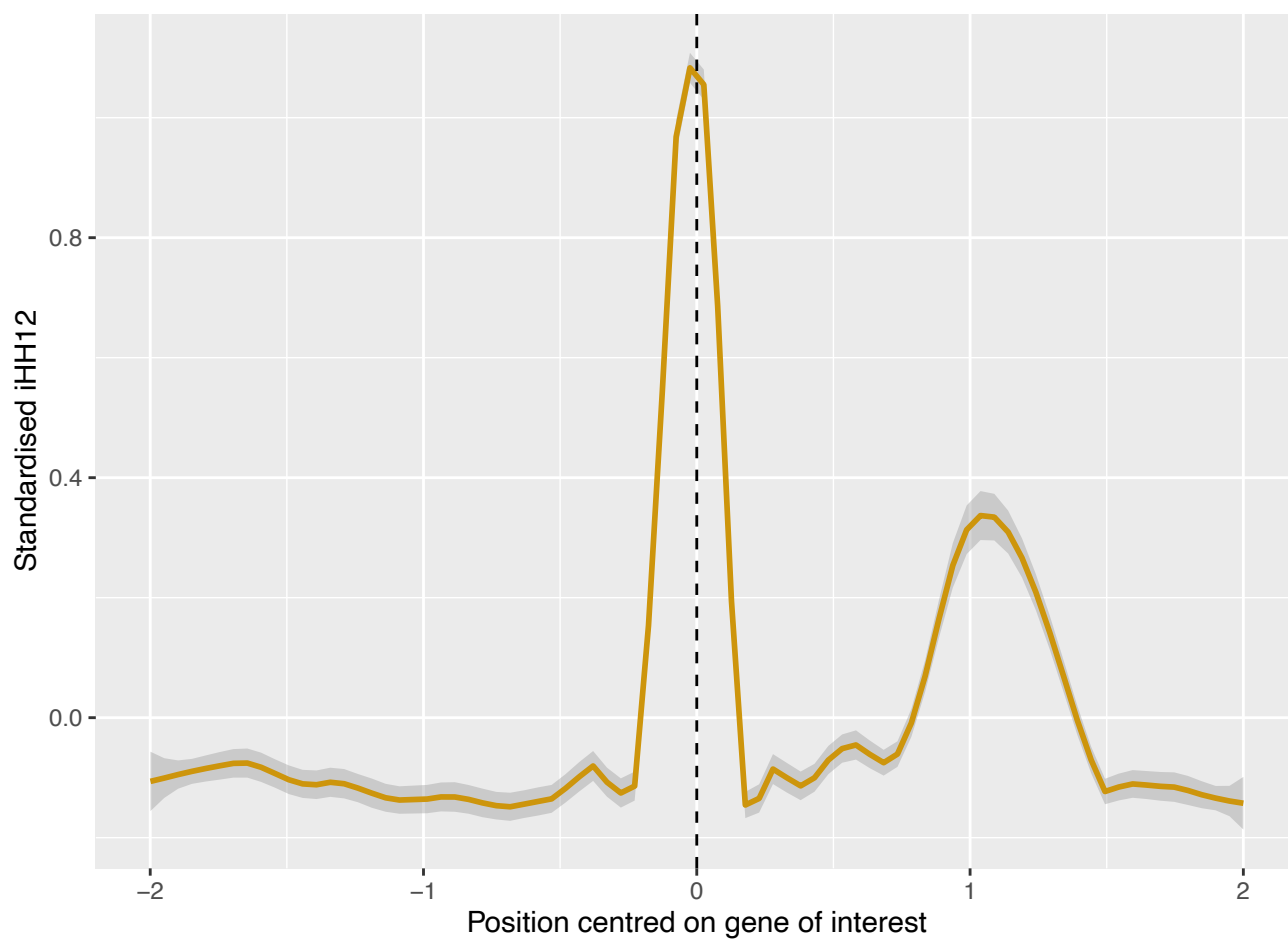

CYP9K1

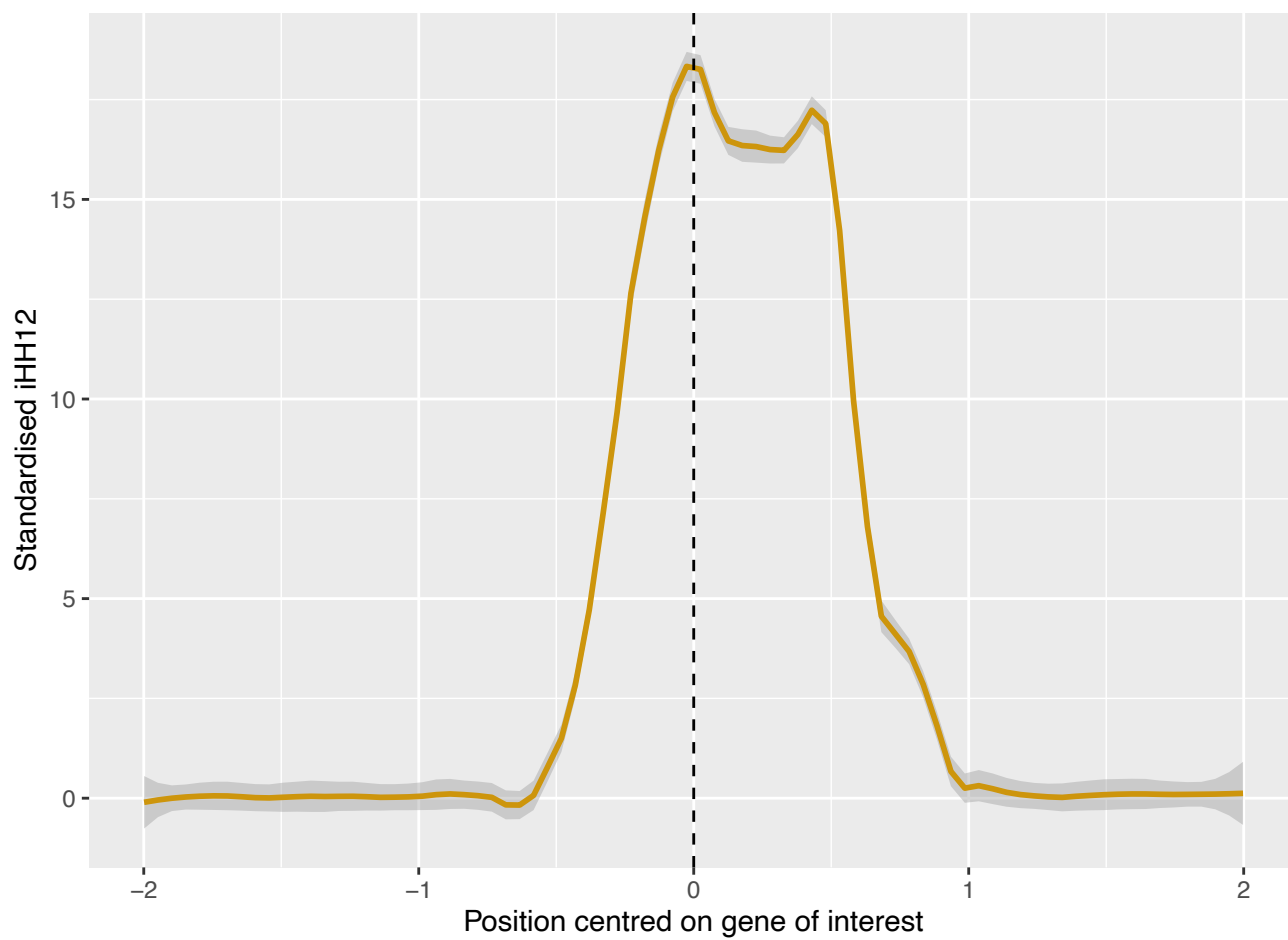

Supplement: Supplementary file 5 — Supplementary Material 5. Supplementary Figure 5: H12 sweeps in the Tengrela (F) population centred on genes of interest. Absolute H12 statistic (y axis) for each resistance-related sweep location in the field-caught Tengrela (F) population. The loci of the genes of interest were taken as the centre point +/- 2Mbp (x axis). The dotted line represents the gene of interest: GSTE2 and CYP9K1. The yellow line represents H12. [file 12864_2026_13109_MOESM5_ESM.pdf]

A.

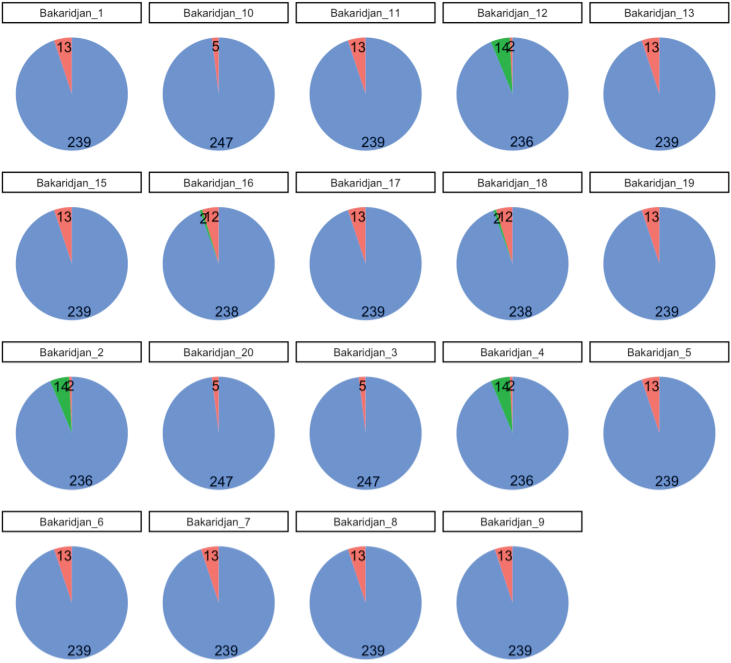

B.

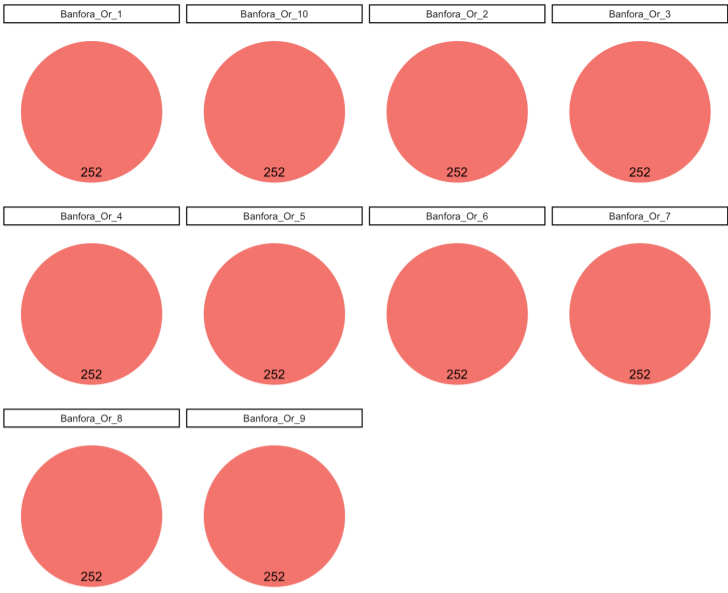

C.

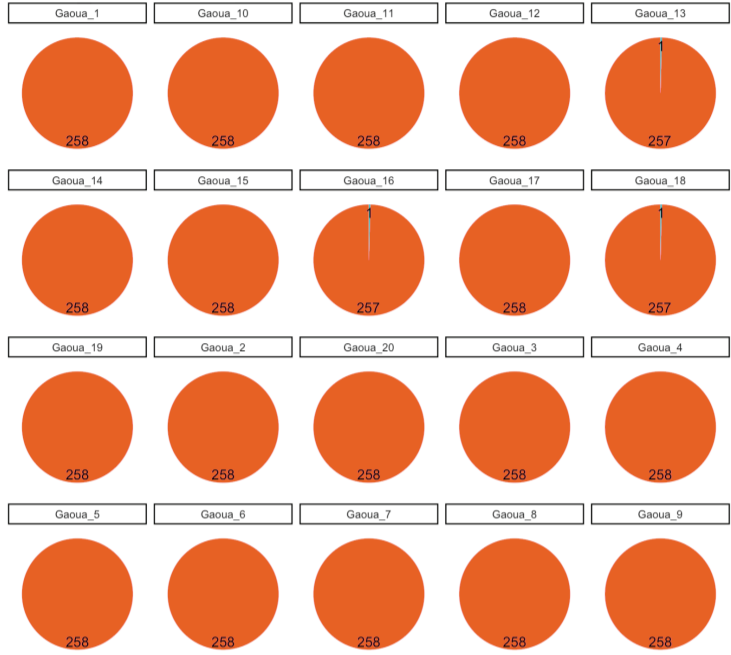

D.

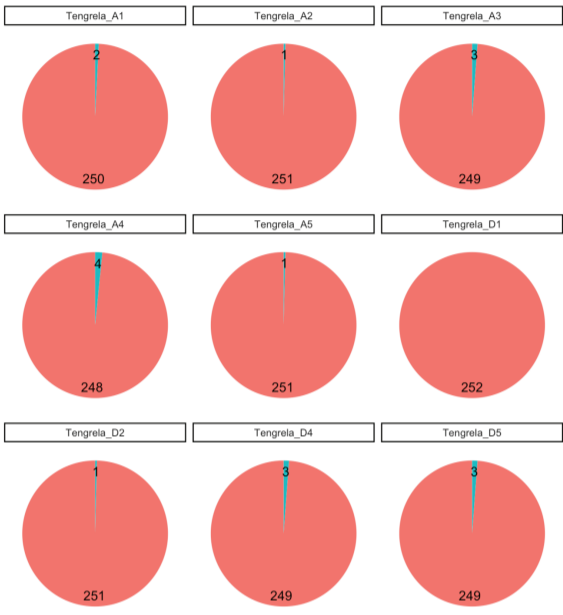

E.

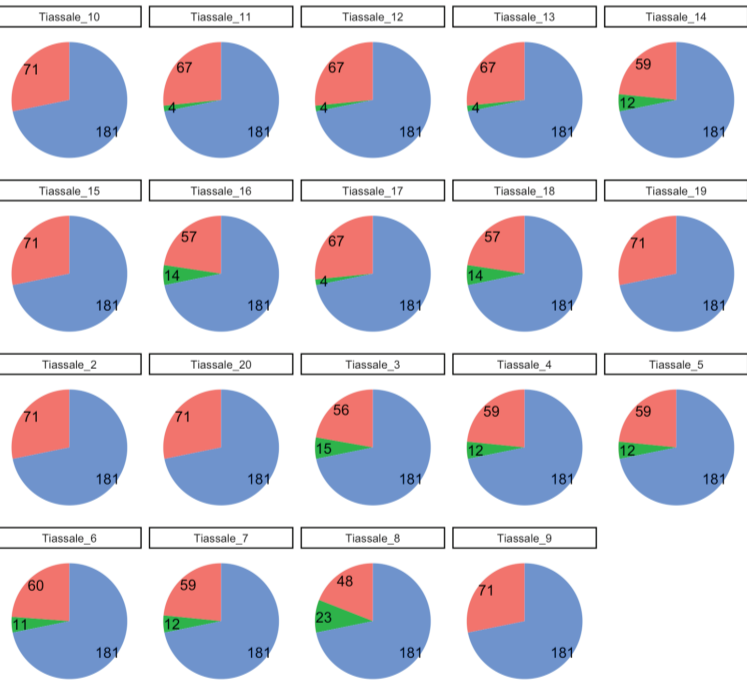

F.

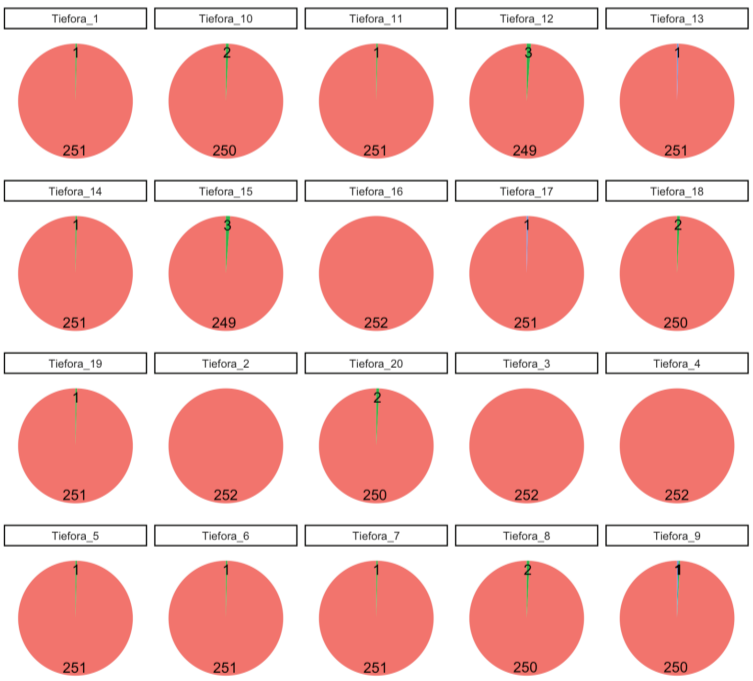

G.

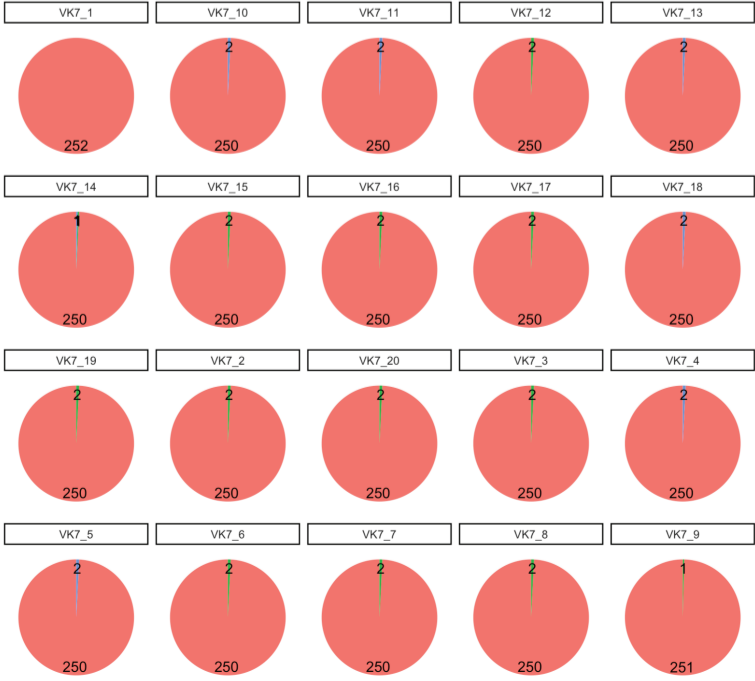

Supplement: Supplementary file 6 — Supplementary Material 6. Supplementary Figure 6: X chromosome ancestry informative markers for each population. Ancestry informative markers used in Ag1000G were extracted from the vcf file and assigned the expected species. A. Bakaridjan_Ag, B. Banfora, C. Gaoua_Aa, D. Tengrela (F), E. Tiassalé_Ag.sl, F. Tiefora and G. VK7. Blue shows An. gambiae markers, pink shows An. coluzzii, orange shows An. arabiensis and green shows heterozygotes. [file 12864_2026_13109_MOESM6_ESM.pdf]

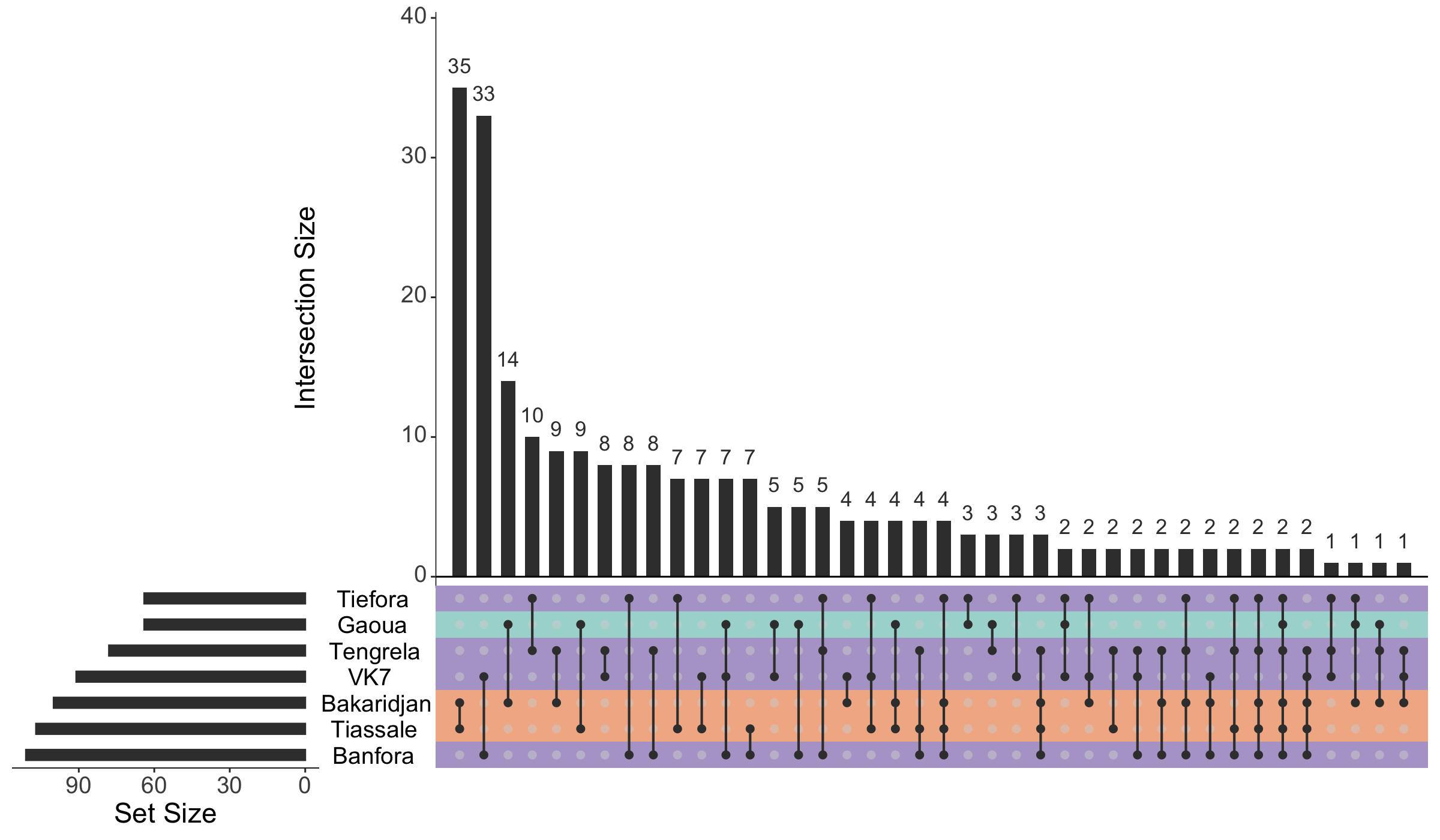

Supplement: Supplementary file 7 — Supplementary Material 7. Supplementary Figure 7: Summary of shared haplotype regions. The number of shared haplotype regions in the top 5 % of windowed ABBA-BABA and bottom 5% of FST statistics across all populations. The intersection size (y) shows the number of regions shared between the populations listed below the x-axis. Each dot connected by a solid line shows the populations with the shared haplotypes. The bar chart to the left shows the total number of putative haplotypes shared for each population. An. coluzzii are purple, An. gambiae orange and An. arabiensis teal. [file 12864_2026_13109_MOESM7_ESM.tif]

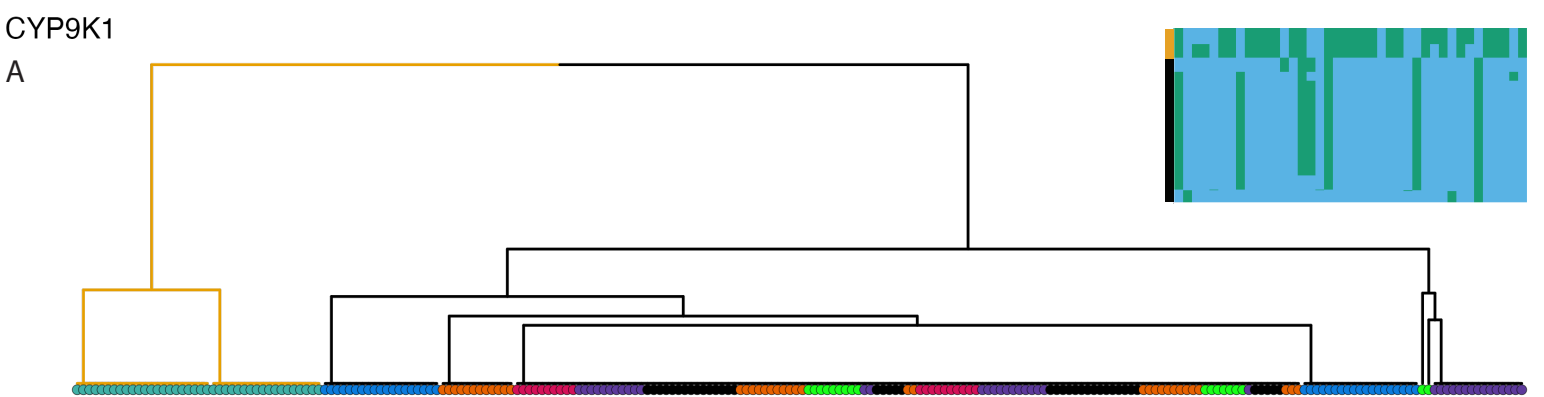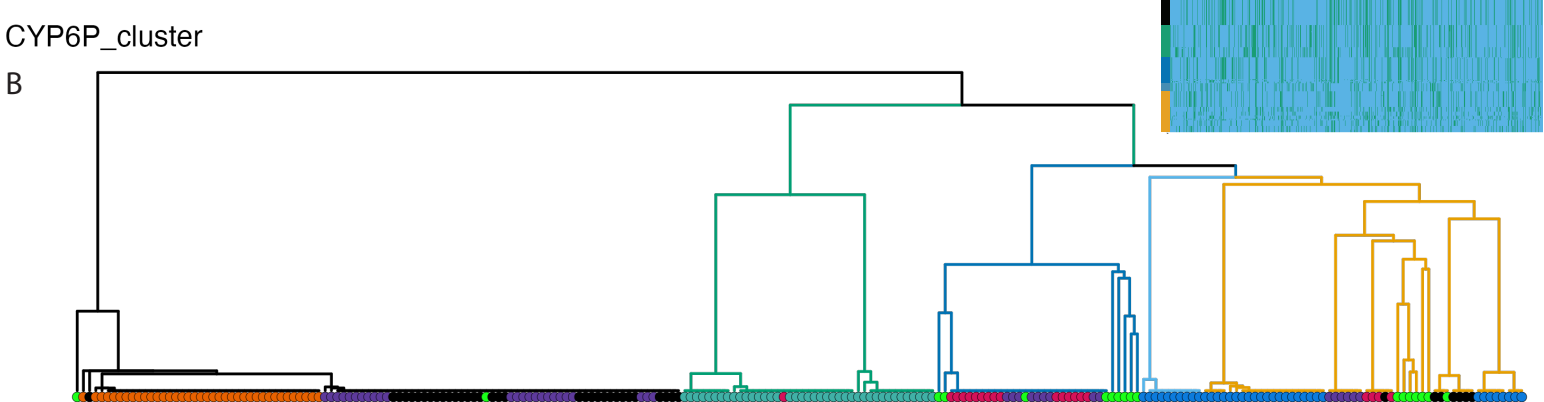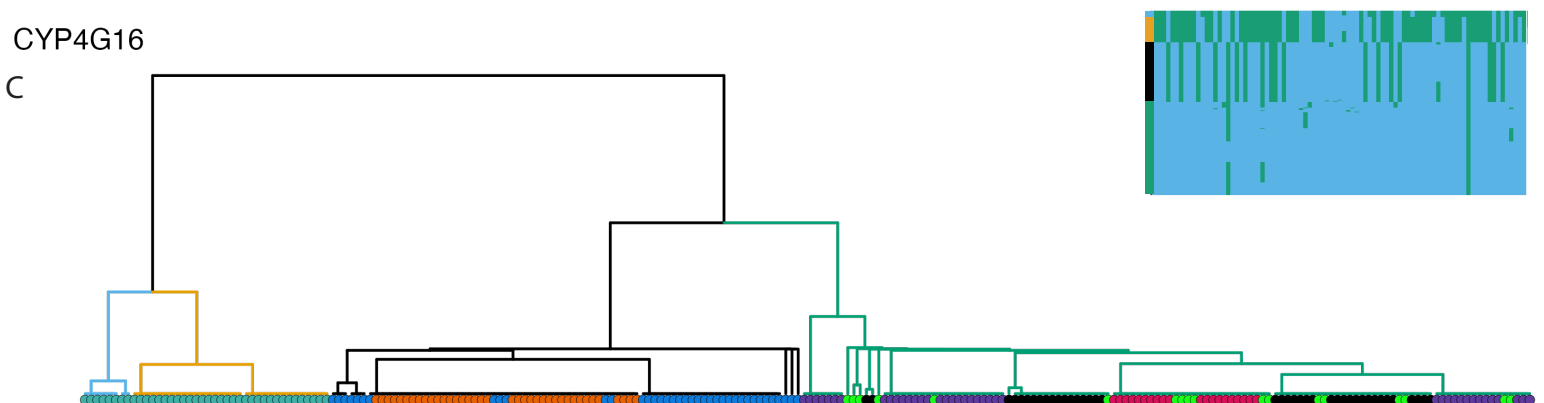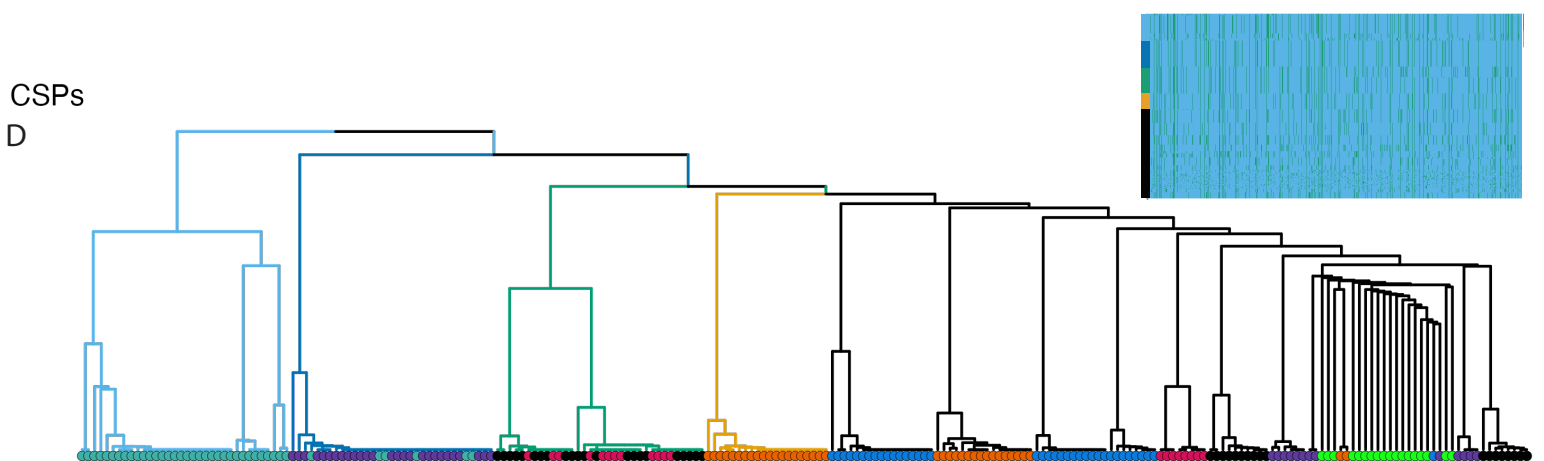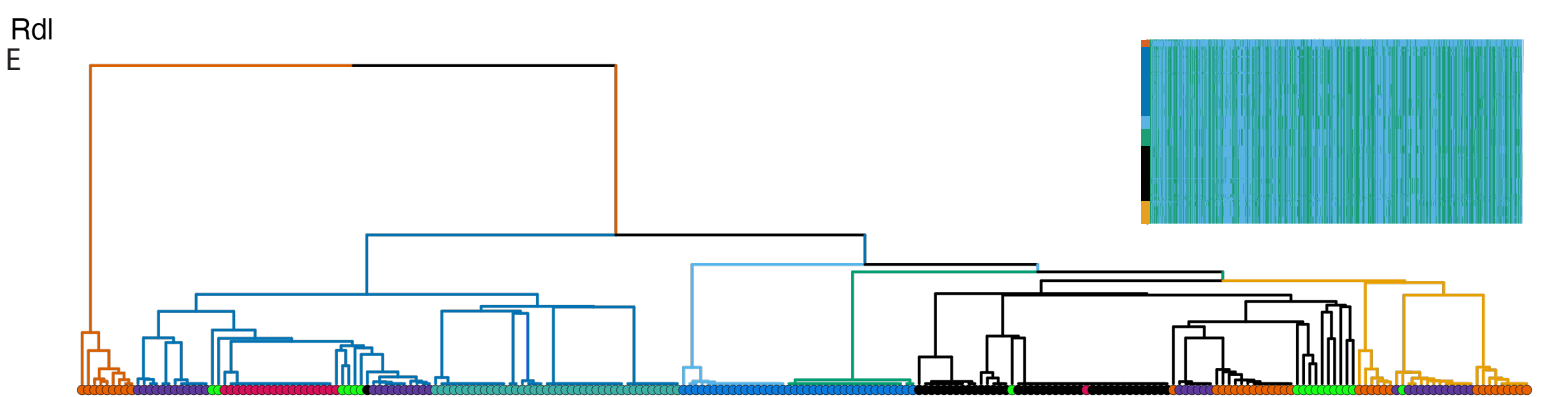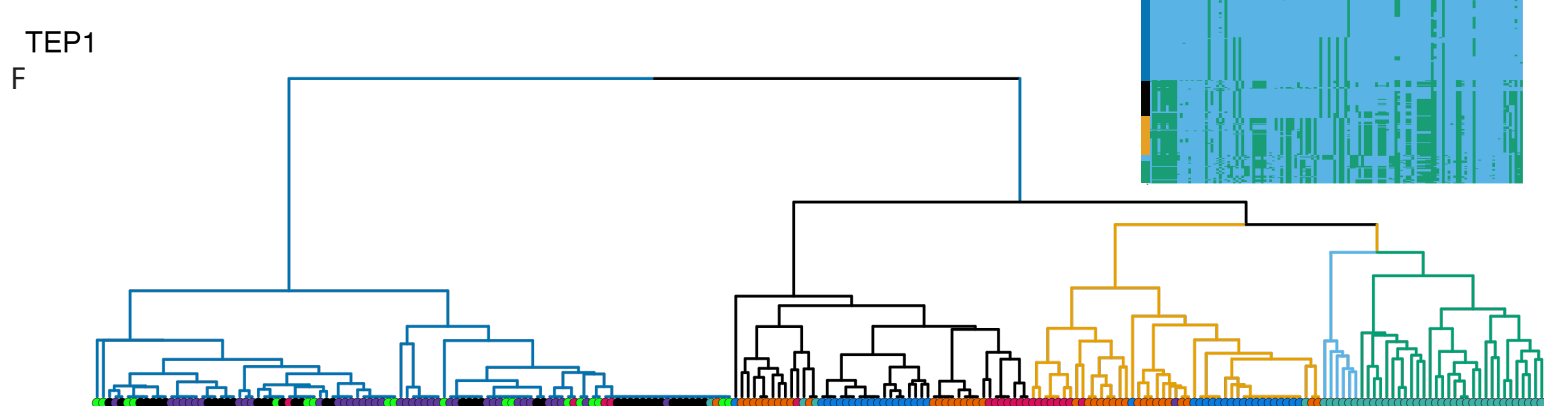

Supplement: Supplementary file 8 — Supplementary Material 8. Supplementary Figure 8: Dendrogram for loci with shared haplotypes. Dendrograms for A. CYP9K1, B. CYP6P cluster, C. CYP4G16, D. CSP cluster, E. Rdl and F. TEP1 branch length represents numbers of SNP differences. Clusters defined by optimal k as determined by a silhouette plot are represented through coloured branches and nodes labelled by species colours. For each, a heatmap showing wt (blue) and derived (green) alleles are shown to give an indication of the diversity within and between clusters. Colours at the side of the heatmaps represent the branch colours. [file 12864_2026_13109_MOESM8_ESM.pdf]

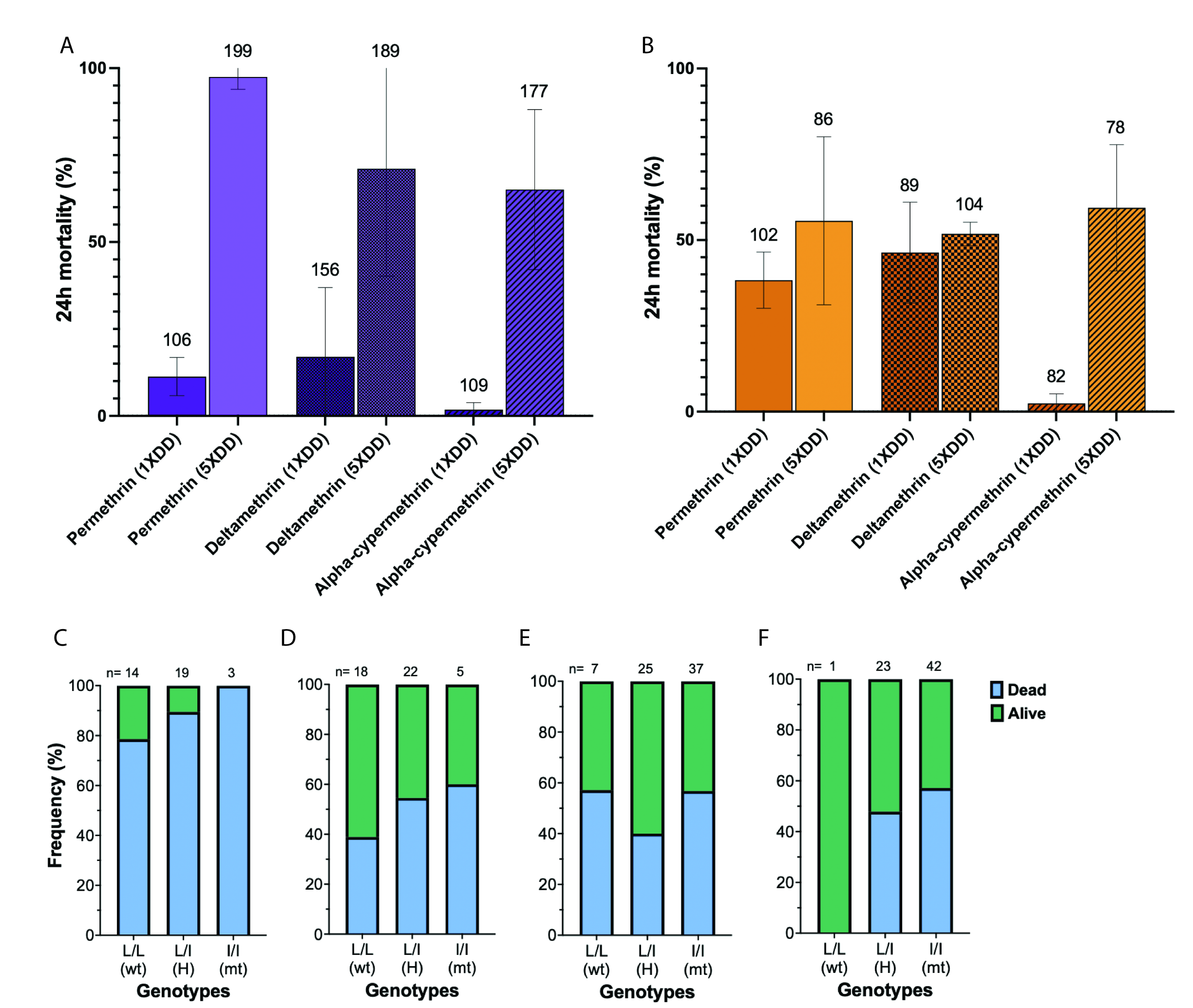

Supplement: Supplementary file 9 — Supplementary Material 9. Supplementary Figure 9: Mortality of Tiefora and Tiassalé_Ag.sl mosquitoes and association testing. A. Mortality of Tiefora and B. Tiassalé_Ag.sl to WHO tubes of 1X and 5X DD of deltamethrin, permethrin and alpha-cypermethrin. C-F. Association study of L207I with resistance to 5X DD using WHO tube tests of three pyrethroids in the C/D. Tiefora for C. permethrin and D. alpha-cypermethrin and E/F. Tiassalé_Ag.sl for E. permethrin and F. alpha-cypermethrin. n shows number of mosquitoes screened, wt = wild-type, H = heterozygous, mt = mutant, no significance, as calculated by a Fisher Exact Test. [file 12864_2026_13109_MOESM9_ESM.tif]

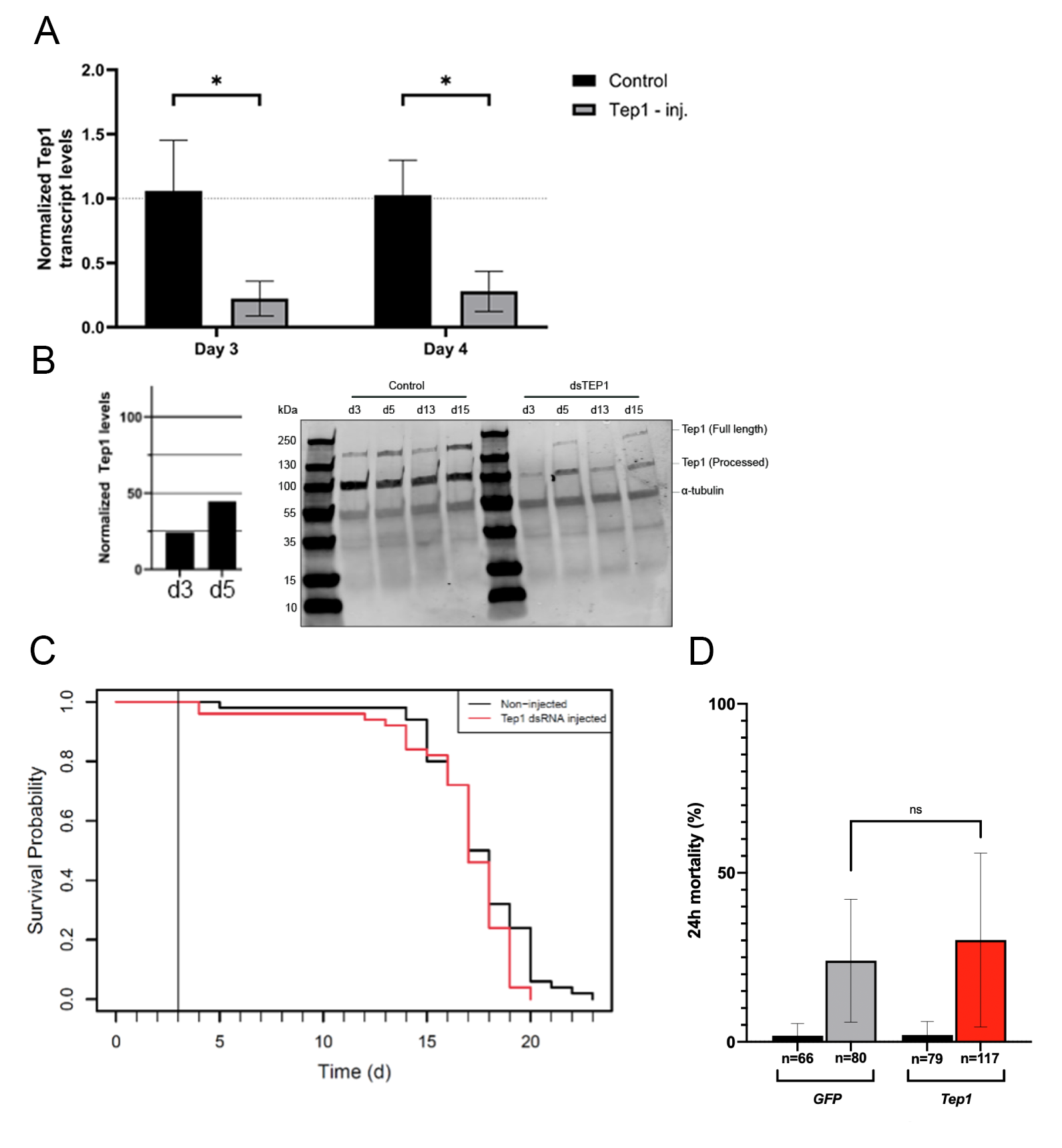

Supplement: Supplementary file 10 — Supplementary Material 10. Supplementary Figure 10: Knockdown of TEP1 has no impact on pyrethroid resistance in Tiassalé_Ag.sl. A. Bar graphs depicting transcript levels (y-axis) of TEP1 after dsRNA injection (grey bars), normalized to the GFP-injected control (black bars) 72- and 96-h post injection. Depicted values are the average of three biological replicates with three technical replicates each, ± SD. Statistical comparison between control and RNAi group were calculated with unpaired t-test: * p < 0.05 B. Bar chart showing comparison of quantified TEP1 (processed) levels between control and TEP1 dsRNA injected mosquitos, after normalization of respective signal to the α-tubulin loading control. Western blot of TEP1 proteins in control dsGFP (left) and dsTEP1 (right) injected mosquitos over a time course, below is the α-tubulin loading control. d3 and d5 refer to day 3- and 5-post-injection. C. Kaplan-Meier-plot comparing the survival of non-injected (black) versus dsTEP1-injected mosquitoes (red). The vertical line represents time of injection. 50 female mosquitos were used per group. D. Observed 24 h mortality of pyrethroid-resistant after 1 h exposure to a control tube or 0.05 % deltamethrin in a WHO-tube assay. Percentage mortality (y axis) for control exposed for each injection group (black), dsGFP (grey) and dsTEP1 (red). Statistical comparison between control and deltamethrin exposed group were calculated with unpaired t-test. Sample size is indicated below bars. All experiments done with Tiassalé_Ag.sl. [file 12864_2026_13109_MOESM10_ESM.tif]
