# Supplementary material for: Multi-omics analysis identifies loci associated with pyrethroid resistance across sister species in the Anopheles gambiae species complex
Source: BMC Genomics. 2026 Jul 7;27:594. doi: 10.1186/s12864-026-13109-8 (PMC13339448; doi:10.1186/s12864-026-13109-8)
Supplement: Supplementary file 3 — Supplementary Material 3. Supplementary Figure 3: Absolute H12 statistic for all populations. H12 statistic (y axis) for the length of each chromosome (x axis) as indicated at the top of each panel. Populations are coloured as previously, and the title of the graph indicates the population. Displayed are the top 5 % of the statistic only, cut-off indicated below. Labels correspond to putative insecticide-resistant associated transcripts in regions of elevated iHS. [file 12864_2026_13109_MOESM3_ESM.pdf]

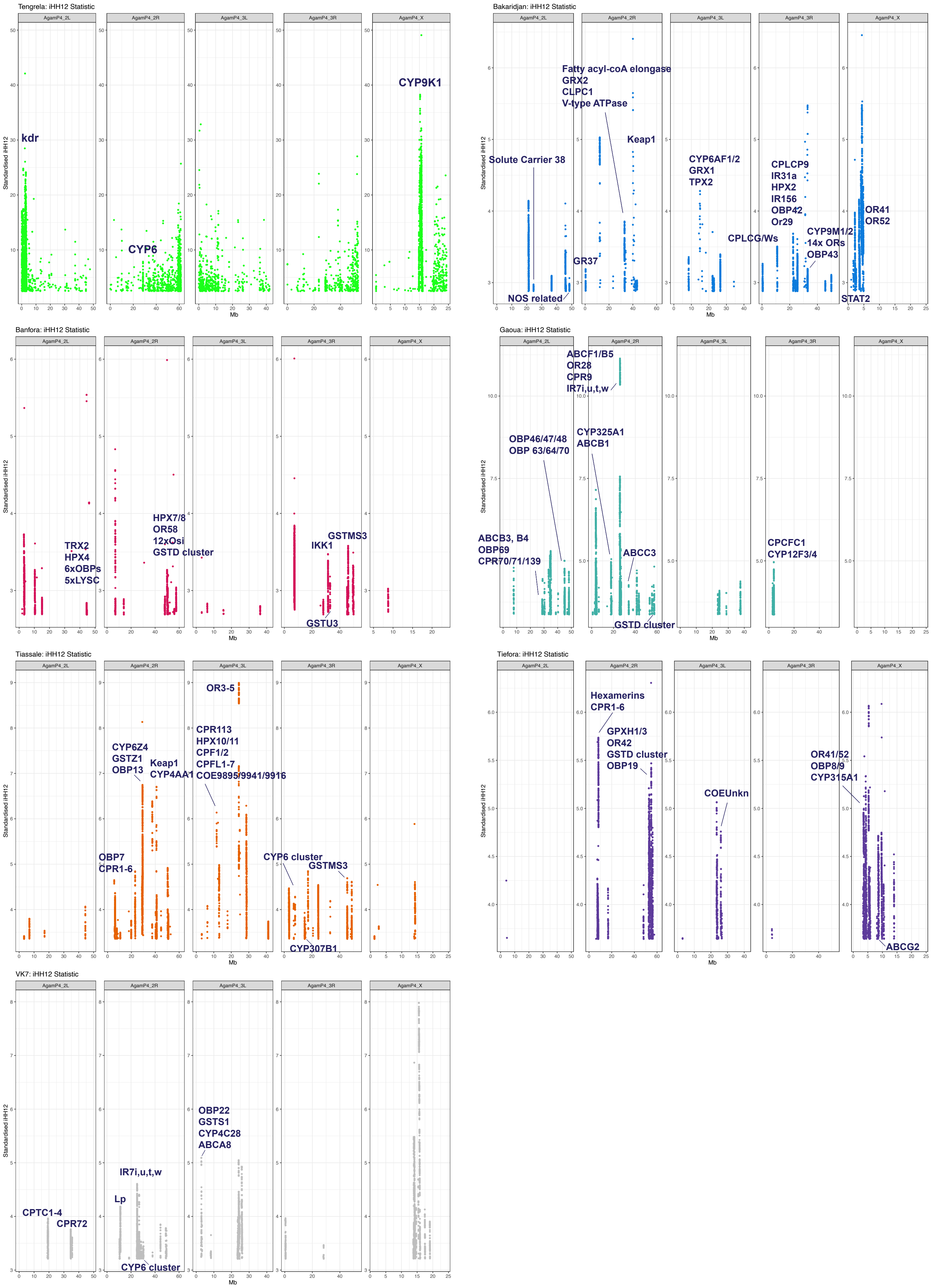

Tengrela: iHH12 Statistic
AgamP4_2L
AgamP4_2R
AgamP4_3L
AgamP4_3R
AgamP4_X
Standardised iHH12
Mb
kdr
CYP6
CYP9K1
Bakaridjan: iHH12 Statistic
Fatty acyl-coA elongase
GRX2
CLPC1
V-type ATPase
Keap1
Solute Carrier 38
GR37
NOS related
CYP6AF1/2
GRX1
TPX2
CPLCP9
IR31a
HPX2
IR156
OBP42
Or29
CPLCG/Ws
CYP9M1/2
14x ORs
OBP43
OR41
OR52
STAT2
Banfora: iHH12 Statistic
TRX2
HPX4
6xOBPs
5xLYSC
HPX7/8
OR58
12xOsi
GSTD cluster
IKK1
GSTMS3
GSTU3
Gaoua: iHH12 Statistic
ABCF1/B5
OR28
CPR9
IR7i,u,t,w
OBP46/47/48
OBP 63/64/70
CYP325A1
ABCB1
ABCB3, B4
OBP69
CPR70/71/139
ABCC3
GSTD cluster
CPCFC1
CYP12F3/4
Tiassale: iHH12 Statistic
OR3-5
CPR113
HPX10/11
CPF1/2
CPFL1-7
COE9895/9941/9916
CYP6Z4
GSTZ1
OBP13
Keap1
CYP4AA1
OBP7
CPR1-6
CYP6 cluster
GSTMS3
CYP307B1
Tiefora: iHH12 Statistic
Hexamerins
CPR1-6
GPXH1/3
OR42
GSTD cluster
OBP19
COEUnkn
OR41/52
OBP8/9
CYP315A1
ABCG2
VK7: iHH12 Statistic
CPTC1-4
CPR72
IR7i,u,t,w
Lp
CYP6 cluster
OBP22
GSTS1
CYP4C28
ABCA8
