# Supplementary material for: Multi-omics analysis identifies loci associated with pyrethroid resistance across sister species in the Anopheles gambiae species complex
Source: BMC Genomics. 2026 Jul 7;27:594. doi: 10.1186/s12864-026-13109-8 (PMC13339448; doi:10.1186/s12864-026-13109-8)
Supplement: Supplementary file 11 — Supplementary Material 11. Supplementary Figure 11: H12 and eQTL information for pyrethroid resistance-related transcripts. Dot plots showing Ag1000G H12 peak regions of interest for insecticide resistance-related transcripts (left y axis) across chromosomes are shown with An. gambiae in pink and An. coluzzii in yellow along with eQTL p-values (right y axis) in grey. For each region of each chromosome, representative SNPs of interest showing increases in normalised read count (y axis) with genotype (x-axis) are shown as box plots with overlayed points. Genotype 0 represents reference allele, 1 heterozygote and 2 derived. Points are coloured by population as shown in the key. SNP locations are given in the following format AgamP4_chromosome:SNP locus. [file 12864_2026_13109_MOESM11_ESM.pdf]

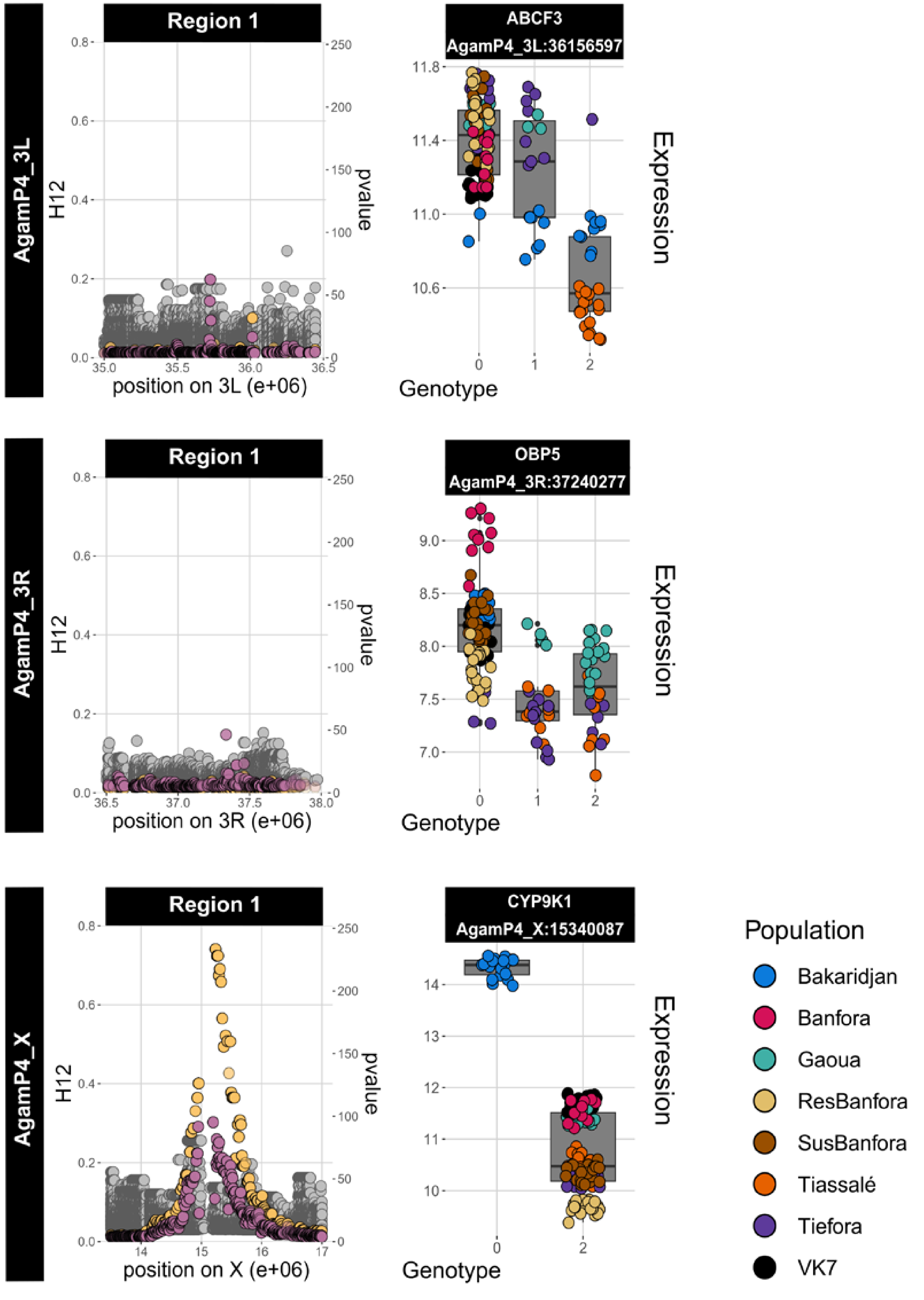

AgamP4_3L
Region 1
H12
pvalue
position on 3L (e+06)
ABCF3
AgamP4_3L:36156597
Expression
Genotype
AgamP4_3R
Region 1
H12
pvalue
position on 3R (e+06)
OBP5
AgamP4_3R:37240277
Expression
Genotype
AgamP4_X
Region 1
H12
pvalue
position on X (e+06)
CYP9K1
AgamP4_X:15340087
Expression
Genotype
Population
Bakaridjan
Banfora
Gaoua
ResBanfora
SusBanfora
Tiassalé
Tiefora
VK7

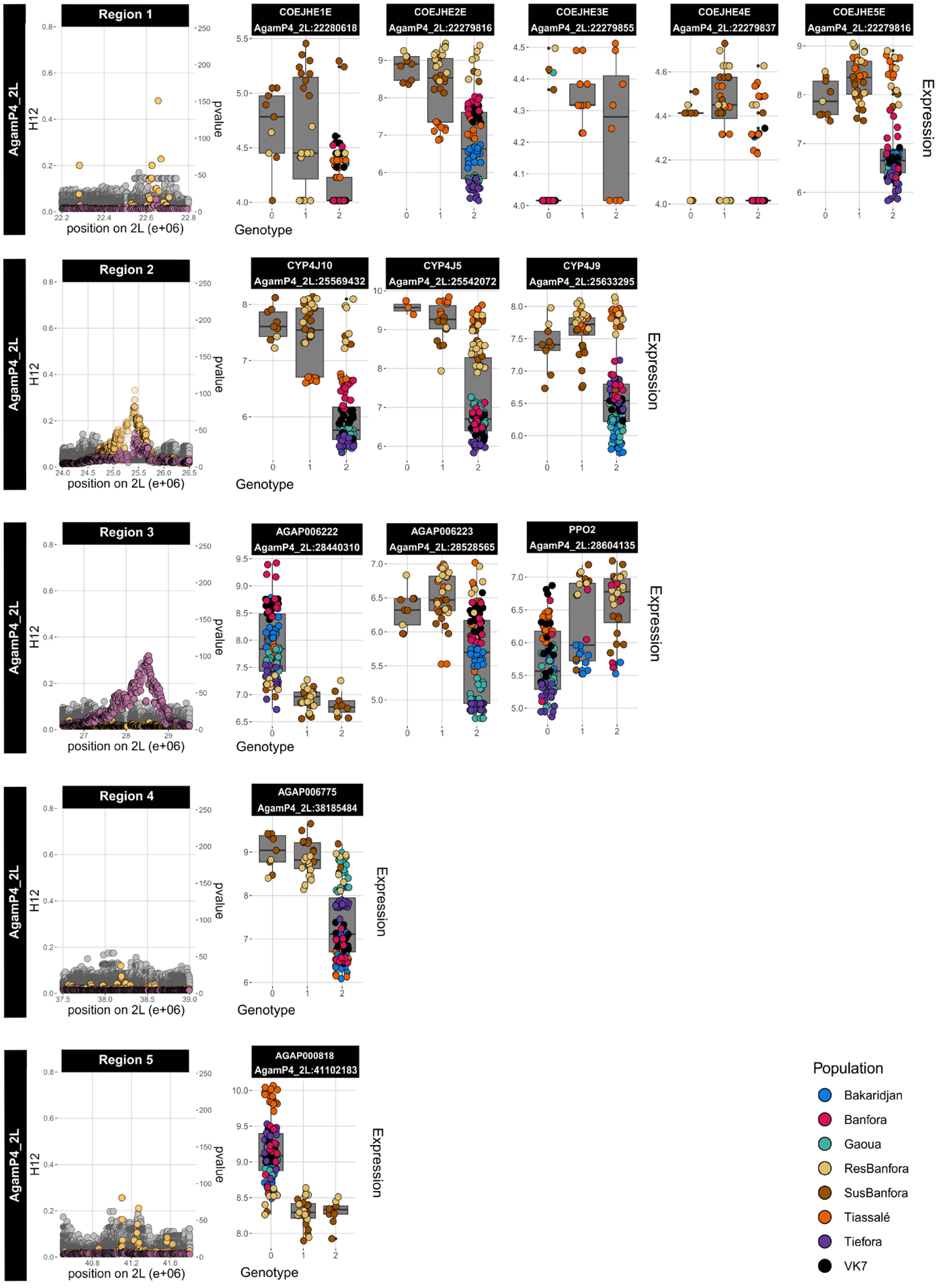

Region 1
COEJHE1E AgamP4_2L:22280618
COEJHE2E AgamP4_2L:22279816
COEJHE3E AgamP4_2L:22279855
COEJHE4E AgamP4_2L:22279837
COEJHE5E AgamP4_2L:22279816
Region 2
CYP4J10 AgamP4_2L:25569432
CYP4J5 AgamP4_2L:25542072
CYP4J9 AgamP4_2L:25633295
Region 3
AGAP006222 AgamP4_2L:28440310
AGAP006223 AgamP4_2L:28528565
PPO2 AgamP4_2L:28604135
Region 4
AGAP006775 AgamP4_2L:38185484
Region 5
AGAP000818 AgamP4_2L:41102183
AgamP4_2L
H12
pvalue
position on 2L (e+06)
Genotype
Expression
Population
Bakaridjan
Banfora
Gaoua
ResBanfora
SusBanfora
Tiassalé
Tiefora
VK7

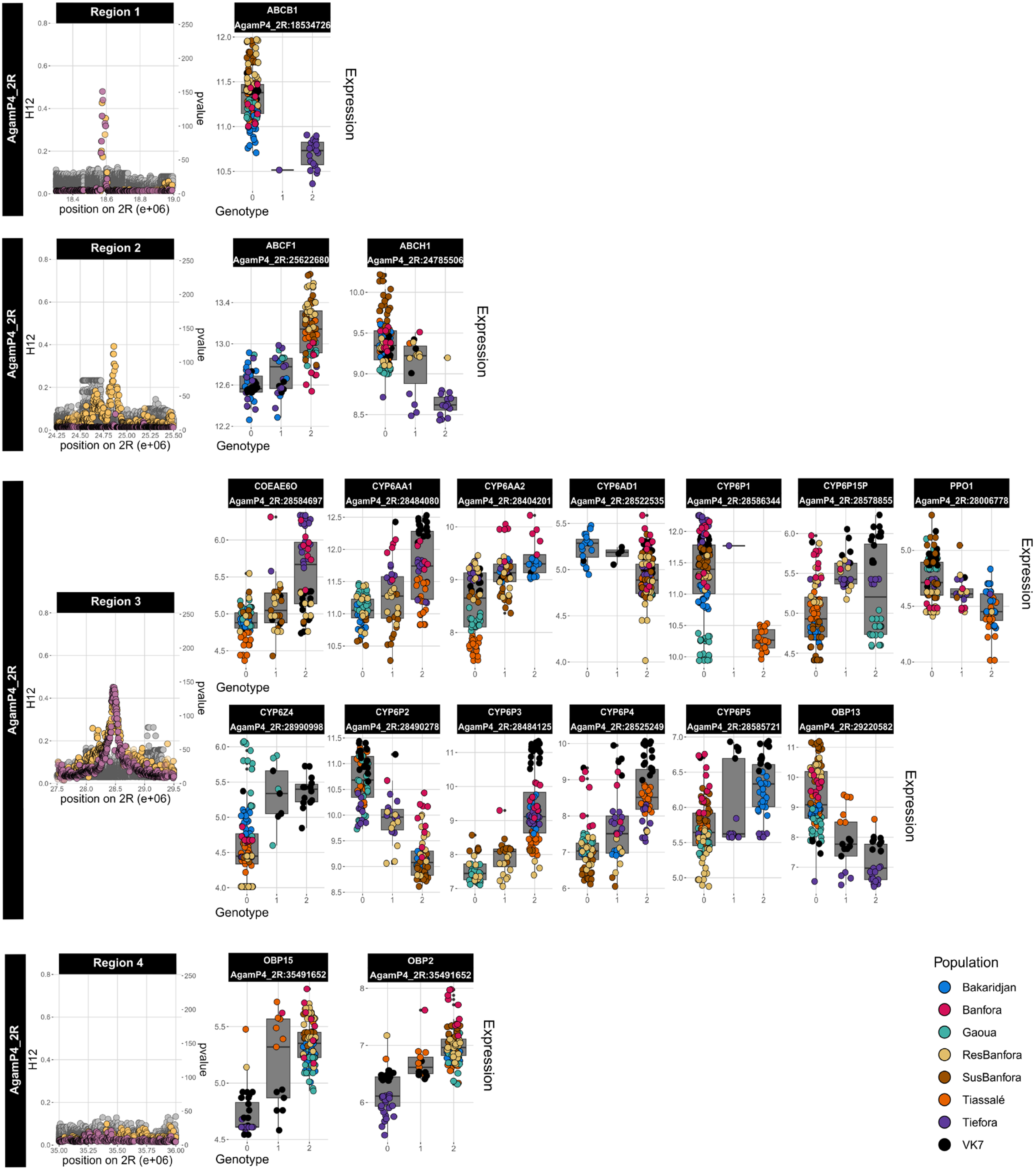

Region 1
Region 2
Region 3
Region 4
AgamP4_2R
H12
pvalue
position on 2R (e+06)
Genotype
Expression
ABCB1 AgamP4_2R:18534726
ABCF1 AgamP4_2R:25622680
ABCH1 AgamP4_2R:24785506
COEAE6O AgamP4_2R:28584697
CYP6AA1 AgamP4_2R:28484080
CYP6AA2 AgamP4_2R:28404201
CYP6AD1 AgamP4_2R:28522535
CYP6P1 AgamP4_2R:28586344
CYP6P15P AgamP4_2R:28578855
PPO1 AgamP4_2R:28006778
CYP6Z4 AgamP4_2R:28990998
CYP6P2 AgamP4_2R:28490278
CYP6P3 AgamP4_2R:28484125
CYP6P4 AgamP4_2R:28525249
CYP6P5 AgamP4_2R:28585721
OBP13 AgamP4_2R:29220582
OBP15 AgamP4_2R:35491652
OBP2 AgamP4_2R:35491652
Population
Bakaridjan
Banfora
Gaoua
ResBanfora
SusBanfora
Tiassalé
Tiefora
VK7
